# Patterns of HIV prevalence among injecting drug users in the cross-border area of Lang Son Province, Vietnam, and Ning Ming County, Guangxi Province, China

**DOI:** 10.1186/1471-2458-5-89

**Published:** 2005-08-24

**Authors:** Don C Des Jarlais, Patrick Johnston, Patricia Friedmann, Ryan Kling, Wei Liu, Doan Ngu, Yi Chen, Tran V Hoang, Meng Donghua, Ly K Van, Nguyen D Tung, Kieu T Binh, Theodore M Hammett

**Affiliations:** 1 Baron Edmond de Rothschild Chemical Dependency Institute, Beth Israel Medical Center, New York City, USA; 2 Abt Associates, Inc., Cambridge, USA; 3 Guangxi Ctr. for HIV/AIDS and Prevention and Control, Nanning, China; 4 Hanoi, Vietnam; 5 Family Health International, Hanoi, Vietnam; 6 Ning Ming County Health Department, Ning Ming City, Guangxi, China; 7 Lang Son Provincial Health Service, Lang Son, Vietnam; 8 General Department of Preventive Medicine and HIV/AIDS Control, Ministry of Health, Hanoi, Vietnam; 9 Hanoi, Vietnam

## Abstract

**Background:**

To assess patterns of injecting drug use and HIV prevalence among injecting drug users (IDUs) in an international border area along a major heroin trans-shipment route.

**Methods:**

Cross-sectional surveys of IDUs in 5 sites in Lang Son Province, Vietnam (n = 348) and 3 sites in Ning Ming County, Guangxi Province, China (n = 308). Respondents were recruited through peer referral ("snowball") methods in both countries, and also from officially recorded lists of IDUs in Vietnam. A risk behavior questionnaire was administered and HIV counseling and testing conducted.

**Results:**

Participants in both countries were largely male, in their 20s, and unmarried. A majority of subjects in both countries were members of ethnic minority groups. There were strong geographic gradients for length of drug injecting and for HIV seroprevalence. Both mean years injecting and HIV seroprevalence declined from the Vietnamese site farthest from the border to the Chinese site farthest from the border. 10.6% of participants in China and 24.5% of participants in Vietnam reported crossing the international border in the 6 months prior to interview. Crossing the border by IDUs was associated with (1) distance from the border, (2) being a member of an ethnic minority group, and (3) being HIV seropositive among Chinese participants.

**Conclusion:**

Reducing the international spread of HIV among IDUs will require programs at the global, regional, national, and "local cross border" levels. At the local cross border level, the programs should be coordinated on both sides of the border and on a sufficient scale that IDUs will be able to readily obtain clean injection equipment on the other side of the border as well as in their country of residence.

## Background

Both injecting drug use and HIV among injecting drug users (IDUs) have become major international public health problems. HIV infection has been reported among IDUs in over 100 countries [1]. Travel by IDUs, particularly along drug distribution routes, appears to be a major mechanism for the spread of both injecting drug use and HIV among IDUs. HIV spread north [2] and south [3] from New York City along the East Coast in the U.S. Stimson [4], and Beyrer and colleagues [5] have reconstructed the spread of HIV among IDUs in South East Asia. Beyrer et al. used molecular epidemiology (mapping the different subtypes of HIV) in their reconstruction. A recent study by Kato and colleagues (2001) found patterns of HIV genetic subtyping consistent with cross-border transmission either from Vietnam to China or from China to Vietnam.

While these regional and country level analyses have great value in understanding the worldwide spread of HIV among IDUs, they have important limitations with respect to HIV prevention efforts. Reducing HIV spread by attempting to disrupt regional and country drug distribution routes may have the unintended consequence of displacing distribution to new routes, leading to additional spread of injecting drug use and HIV among IDUs.

Successful prevention efforts will be greatly facilitated by more detailed understanding of the spread of injecting drug use and transmission of HIV among IDUs within smaller geographic areas. Understanding of HIV transmission across international borders is particularly important, as few HIV prevention programs are coordinated across such borders. We present here data on injecting drug use and HIV among IDUs in the adjacent border provinces of Lang Son, Vietnam and Guangxi, China. HIV among IDUs was noted in this area in 1996 [6] and since then there has been substantial transmission among IDUs in both provinces. The present situation shows a clear geographic pattern, with the potential for additional spread across the border between the provinces and within each of the provinces.

**Figure 1:**
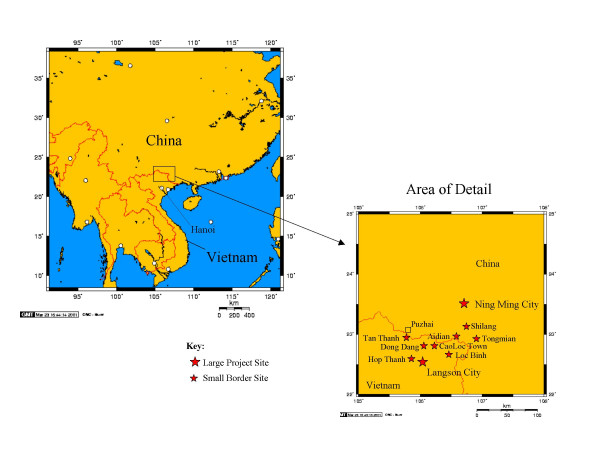
Geographic Setting and map of project sites.

The data reported here were collected as part of baseline surveys of IDUs conducted before implementation of a cross-border HIV prevention intervention in Lang Son Province, Vietnam and-Ning Ming County, Guangxi Province, Vietnam [7]. Figure 1 shows a map of the area, with the project sites – in Lang Son Province and in Ning Ming County.

There is considerable official and entirely legal movement of commercial goods across the border in both directions. There are also semi-official and informal crossing points and pathways through the hills that permit local residents to cross the border with little or no regulation. Crossing the border is a regular aspect of life for many people who live near the border, for example, to attend market days in the larger villages. Drug dealers cross the border to sell drugs and drug users also cross to obtain drugs of higher purity and at better prices. The frequency of border crossing can vary, influenced by current price and purity of heroin, the ebb and flow of law enforcement activity and, factors such as the outbreak of severe acute respiratory syndrome (SARS) in China.

There is also substantial trade and migratory employment in the region. Lang Son City, Aidian, and Puzhai (near Pingxiang) are bustling centers of legitimate cross-border trade, as well as drug trafficking and sex work. Many people cross the border daily and seasonally to find work and many are employed as porters in the cross-border trade. The region is home to many ethnic minority groups (e.g., Zhuang, Tay, Nung), some of whom live on both sides of the border (for example, the Zhuang in China and the Tay in Vietnam are the same ethnic group but are known by different names in the two countries). There is frequent intermarriage across the border, although this might be illegal and might lead to loss of nationality. Kinship ties, like migratory employment and trade, result in additional cross-border movement.

## Methods

Data collection methods for the IDU surveys reported on here were essentially parallel in Ning Ming County and Lang Son Province, with some variation in the community-based subject recruitment strategies used. The availability of large known drug use gathering places and of officially registered IDUs in Lang Son permitted greater use of probability-based methods in Vietnam, while there was more reliance on peer recruitment in China.

### Subject recruitment – China

In Ning Ming County, a modified snowball/peer recruitment technique was used. The project peer educators sent recruiting letters to IDUs they knew personally, inviting them to come to a project center and participate in the survey. The IDUs who came to project centers for interviews were encouraged to recruit 2–3 additional participants. The research participants received 20 Chinese yuan (approximately $2.50) for the interview, 5 yuan for each additional male respondent recruited, and 10 yuan for each additional woman respondent recruited. The eligibility criteria were a minimum of 18 years of age and recent (in the past 6 months) drug injection.

### Subject recruitment – Vietnam

Approximately one-half of the sample was based on individuals initially selected from the lists of known IDUs in the project sites. The other half was based on participants initially selected from IDUs present at gathering or shooting places mapped by project staff as part of the initial project implementation. For the half of the sample based initially on registered lists, 10 clusters of 25 individuals each were selected by probability proportional to size (PPS) from the lists of IDUs in each commune. Then four IDUs were picked at random from each selected cluster and these referred others until the quota for the commune was reached.

For the portion of the sample selected initially at IDU gathering or shooting places, sample quotas for these places were determined by PPS based on the numbers of individuals observed at these places during the mapping phase. The interview team then revisited the selected places and chose four individuals at random from among those present at each place at that time (who were not necessarily those present during the mapping phase).

The Vietnamese participants were paid 30,000 Vietnamese dong (approximately US$2) for participating in the interview and HIV test.

### Informed consent

In Vietnam, an oral informed consent was obtained for participation in the study, with the interviewer certifying that oral consent had been obtained. This procedure was requested by the Institutional Review Board of the National AIDS Standing Bureau in order to provide more assurance of confidentiality to prospective participants. In China, standard signed informed consents were obtained from all participants. Unique codes were constructed for each participant based on numeric date of birth and several letters representing, for example, the first letter of the mother's family name. (Construction of the record number was slightly different in the two countries.) The objective was to have a unique identifier that the participant could readily reconstruct if he or she lost the project participation card.

### Questionnaire

A structured instrument was used for the interviews, based on version 2b of the questionnaire being used in the World Health Organization's Drug Injection Study, Phase II [8]. Trained interviewers, primarily staff of the local health departments, conducted the interviews. The questionnaire covered demographics, drug use, injection and sexual risk behavior, HIV testing history, HIV and hepatitis knowledge, and cross-border travel patterns. A question on the number of times the subject had crossed the border in the 6 months prior to the interview was included.

There are many factors which could influence the "ease/difficulty" in crossing an international border, including distance to the border, cost of transportation, time needed to reach the border, and the need to have official papers for crossing. It was not practical to measure all such factors. Instead, used simple physical distance (in kilometers) to the nearest border point. This gave five distance categories in Vietnam and three distance categories in China.

The baseline survey was conducted in July 2002 in Vietnam and between July and September 2002 in China.

### HIV testing

The survey included HIV antibody testing. Participants were given pre-test counseling and post-test counseling at local health centers. Blood was drawn at the time of the interviews by trained phlebotomists from local health departments. Participants were given a card with their unique identifier and returned to the local health center to receive their test results using this identification number. Indeed, they could only retrieve their results by using this number since blood samples were not otherwise labeled. In China, testing was by double ELISA (Vironostika HIV- Uni-form, Organon (Holland)) with confirmation of initial HIV-positive results by Western Blot (Genelabs Diagnostics). All testing was conducted at the laboratory of the Guangxi Center for HIV/AIDS Prevention and Control in Nanning. In Vietnam, testing was performed at the laboratory of the Lang Son Provincial Health Services using the Serodia SFD screening test (Biorad {France}) and double ELISA (Genescreen, Biorad (France); Vironostika, Organon [9]). This is the official protocol of the Ministry of Health in Vietnam and the Lang Son laboratory is authorized to provide HIV testing according to this protocol by the Ministry of Health.

### Data analysis

Data were entered and data sets were prepared in EpiInfo, version 6.04 by staff of the Guangxi Center for HIV/AIDS Prevention and Control and the National AIDS Standing Bureau of Vietnam (which has since been merged into the General Department of Preventive Medicine and HIV/AIDS Control of the Ministry of Health). The data were analyzed at Beth Israel Medical Center and Abt Associates Inc. using the Statistical Analysis System, Version 8.2 (SAS, Inc., Cary, NC). Chi square tests were used to assess bivariate relationships among categorical variables. Multi-collinearity problems precluded multivariate analyses.

### Ethical review

The study was reviewed and approved by the institutional review boards (IRBs) of the following institutions: Guangxi Center for HIV/AIDS Prevention and Control, the National AIDS Standing Bureau of Vietnam, Abt Associates Inc., and Beth Israel Medical Center.

## Results

Table 1 shows selected demographic characteristics of the IDU subjects recruited in China and Vietnam. In both provinces, the subjects were primarily young males who had never been married. Although there are known to be female IDUs on both sides of the border, the project has had difficulty inducing women to participate in the interventions and in recruiting them for the surveys. Over two-thirds of the subjects in China and one-half in Vietnam belonged to ethnic minority groups (primarily Zhuang in China and Tay and Nung in Vietnam). The ethnic minority subjects tended to live closer to the border. Table 2 and 3 shows the percentages of ethnic minority subjects in the different sites by distance to the border (in kilometers).

**Table 1:** Characteristics of the Baseline IDU Samples

**Characteristic**	**China (n = 294)**	**Vietnam (n = 348)**
Male	90%	99%
Age 21–30	68%	72%
Ethnic Minority^1^	72%	51%
Married	26%	32%
Never Married	68%	63%
HIV Prevalence	16%	46%
Previous HIV test	7%	34%

1. Defined as a respondent not of Han ethnicity in China and not of Kinh ethnicity in Vietnam.

**Table 2:** Distance from border and ethnic minority percentage among injecting drug users in border area

China	**Ethnic minority**		**P Value**
	**Yes**	**No**	
**Distance from the Border**			
0 km	33 (83%)	7 (18%)	P = .1118
19 km	15 (83%)	3 (17%)	
57 km	162 (69%)	73 (31%)	

**Table 3:** Distance from border and ethnic minority percentage among injecting drug users

**Vietnam**	Ethnic minority		P Value
	**Yes**	**No**	
**Distance from the Border**			
1 km	29 (73%)	11 (28%)	P = .0017
3 km	20 (57%)	15 (43%)	
12 km	29 (62%)	11 (38%)	
13 km	29 (64%)	16 (36%)	
14 km	86 (43%)	113 (57%)	

**Figure 2:**
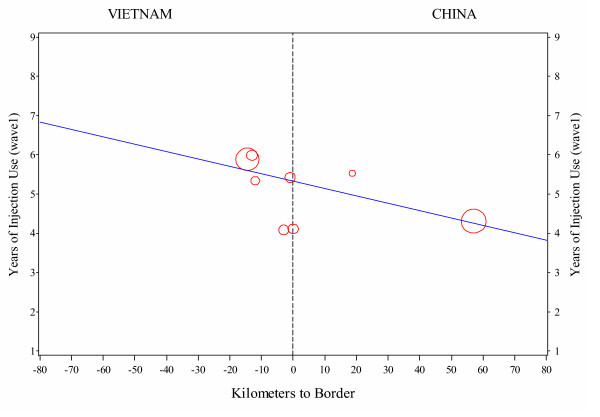
Years of injection use against distance to the border.

Figure 2 shows the relationship between mean number of years injecting drugs and the site's distance from the border. There is a general gradient of decreasing mean years of drug injecting running from the site farthest from the border on the Vietnamese side (Lang Son City) to the site farthest from the border on the Chinese side (Ning Ming City). This gradient was statistically significant, p = -0.002. The estimated slope was -0.02, so the average number of years of injection use decreased by about a week (0.02 of a year) for each kilometer in the Chinese direction. This was based on a weighted linear regression model fitted to the mean number of years used in a site and distance to the border of the site. The weights were based on the sample size at each site.

**Figure 3:**
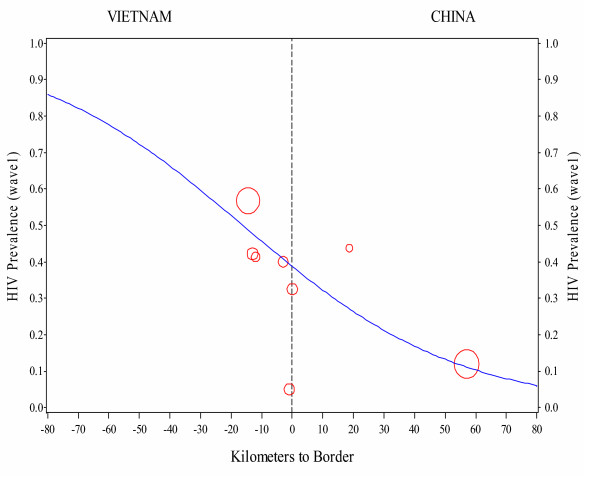
HIV prevalence against distance to border. Gradient plots at baseline.

Figure 3 shows the HIV prevalence by distance to the border for the sites in Vietnam and China for the baseline data. There is a strong gradient of decreasing prevalence from the largest Vietnamese site and the one farthest from the border (Lang Son City) to the largest Chinese site and the one farthest from the border (Ning Ming City). Only one site- (Tan Thanh in Vietnam) does not fall along this gradient. Overall baseline seroprevalence among IDUs was 46% in Lang Son and in 17% Ning Ming. This gradient was statistically significant, p < 0.001. The estimated odds ratio was 0.97, so the odds of an individual being HIV positive decreased by 2–3% for each kilometer in the Chinese direction. This relationship between the proportion of subjects in a site testing positive and distance to the border was modeled using logistic regression.

Table 2 shows factors related to crossing the border (in the 6 months prior to the interview) among the subjects in Vietnam and China. Overall, 10.6% of subjects in China and 24.5% of subjects in Vietnam reported having crossed the border in the six months prior to the interview. Being a member of an ethnic minority group and living closer to the border were strongly related to crossing the border in both countries. In Vietnam, being HIV seropositive was negatively related to crossing the border, while in China being HIV seropositive was positively related to crossing the border (although this relationship did not reach statistical significance, p = .12). Being a member of an ethnic minority group was related to being HIV seropostive in China (19% HIV seropositive among ethnic minority subjects versus 10% seropositive among ethnic majority subjects, (p < 0.05). Ethnicity was not related to HIV status in among the Vietnamese subjects.

## Discussion

In this report, we present data on injecting drug use and HIV infection among IDUs in Lang Son province, Vietnam and Ning Ming County, Guangxi Province, China from a baseline survey conducted before implementation of a peer-based cross-border HIV prevention intervention.

Several limitations should be noted. First, we were working with cross-sectional data, where longitudinal data from the initial spread of injecting drug use in the area would have certainly been preferable. Second, there are measurement and sample size limitations. It would have been helpful to have a measure of ease of travel to the border rather than simple physical distance to the border. Also it would have been helpful to have sufficiently large sample sizes so that the relationship between being a member of an ethnic minority group and being HIV seropositive could be examined within individual geographic sites. Despite these limitations, there are very clear patterns in the data.

There are similar gradients for mean length of injecting history and baseline HIV prevalence running in descending order from the Vietnamese site farthest from the border to the Chinese site farthest from the border. These patterns are consistent with the theory that both the practice of drug injection and the prevalence of HIV infection among IDUs spread from Northern Vietnam to Southern China along a major heroin trans-shipment route [5]. The patterns in our data suggest that, in some circumstances, it may be possible to reconstruct histories of the diffusion of injecting drug use and HIV among IDUs using cross-sectional data.

There is clearly a potential for further cross-border transmission of HIV in both directions. Our discussions with the peer educators in the cross-border HIV prevention project suggest three primary reasons for these IDUs crossing the border: 1. Obtaining higher quality/lower priced drugs, 2. Avoiding police pressure on drug injectors, which can be unpredictably variable and involves large-scale periodic crackdowns, and 3. Personal factors, such as migratory employment, commerce, and family ties. It would appear to be very difficult to reduce these reasons for IDUs crossing the border.

Because of the possibility of arrest, IDUs who cross the border are unlikely to carry needles and syringes with them, even if they are crossing the border at places without any supervision or inspection.

Prevention of risky injections among border crossing IDUs will require very good supplies of sterile injection equipment on both sides. If IDUs who crosses the border cannot readily access sterile injection equipment on both sides of the border, then their fellow IDUs will need to have sufficient supplies of sterile injection equipment for use by themselves and the IDUs who cross the border. This will require large-scale safer injection programs on both sides of the border.

The majority of subjects in this study belong to ethnic minority groups, primarily Zhuang in China and Tay and Nung in Vietnam. Ethnic minority IDUs were also overrepresented among the border crossers. The issues of ethnic minority status, injecting drug use, and HIV infection deserve much more research and policy development. Ethnic minority IDUs are more likely to be infected with HIV in many places, from African-American and Latino/a IDUs in New York City [10] to Roma in Eastern Europe [11] to First Nations in Vancouver [12] to Vietnamese in Australia [13] to Manipuris in India [14]. As noted above, there was a strong relationship between ethnic minority status and HIV serpositivity in Ning Ming (OR = 5.08 (95% CI 1.41,18.26, p = .013) [15]. Social stigmatization of ethnic minority communities may make them more vulnerable to illicit drug use, including injecting drug use. Employment discrimination against ethnic minority communities may increase the extent to which drug distribution occurs in these communities, and to which drugs are transported by minority community members. Persons belonging to ethnic minority groups also may have important factors facilitating international travel, such as social support systems and persons that speak the same language on the other side of international borders.

## Conclusion

The data presented here illustrate many of the factors in the international diffusion of HIV among IDUs at modest geographic scale. (There is a total distance of 71 kilometers between the two most distant sites in the study). These include gradients of length of injecting drug use and HIV seroprevalence across the international border, border crossing by IDUs and its association with HIV infection for those crossing from China into Vietnam, overrepresentation of ethnic minority persons among the border crossers. Both injecting drug use and HIV among IDUs are already well established among IDUs on the Vietnamese side of the border and injecting drug use is well established on the Chinese side of the border. HIV is present among IDUs on the Chinese side of the border, but at lower seroprevalence levels than in Lang Son.

There are multiple reasons that people cross the border in this area, and it would not appear to be possible to stop IDUs from crossing the border or from injecting drugs across the border. Thus, HIV prevention goals must include increasing the safety of injections among border crossers (as well as reducing risk behavior among the IDUs who do not cross the border). This will require coordinated HIV prevention that increases the likelihood that IDUs will inject safely on both sides of the border. Such a program has been implemented in Lang Son and Guangxi provinces. It includes peer outreach, increased access to sterile injection equipment through syringe distribution and exchange and a pharmacy voucher program. IDUs may exchange used injection equipment for new needles/syringes or for vouchers that can be redeemed at local pharmacies for needles/syringes, sterile water, and condoms. IDUs may also directly receive new needles/syringes or pharmacy vouchers even if they do not return used equipment. The program also includes large-scale collection and safe disposal of used needles/syringes, general community education about drugs and HIV, and social support for people living with HIV or AIDS [7].

Reducing the international transmission of HIV among injecting drug users will require programs at the global, regional, national, and "local cross-border" levels. The local cross border programs will need to be coordinated on both sides of the border and on a sufficient scale that IDUs who cross the border will be able to readily obtain clean injection equipment on the other side of the border. The cross-border HIV prevention project currently being implemented in Lang Son Province and Ning Ming County, Guangxi offers an example of how such a coordinated approach can be implemented. Evaluation data being collected in Lang Son and Ning Ming will be used to gauge the effectiveness of the interventions.

## Competing interests

The author(s) declare that they have no competing interests.

## Authors' contributions

TH conceived of the study, the study design and coordination and assisted in the drafting of the manuscript. DCD participated in the design of the study and drafted and edited the manuscript. TH and DCD supervised the data analysis.

PF, PJ and RK performed the statistical analysis and participated in its design and coordination and participated in the writing and review of drafts of the manuscript.

WL, YC & DM supervised the implementation of the project and data collection and processing for the Chinese sites, and participated in the writing and review of drafts of the manuscript.

DN, TVH, LKV, NDT & KTB supervised the implementation of the project and data collection and processing for the Vietnamese sites, and participated in the writing and review of drafts of the manuscript.

All authors read and approved the final manuscript.

**Table 4:** Factors associated with crossing the border among injecting drug users in the China-Vietnam border area

**Vietnam**	**Crossed borders**		**P Value**
	Yes	No	
**Ethnic Minority**			
Yes	56 (31%)	125 (69%)	P = .0015
No	27 (16%)	138 (84%)	
**HIV**			
Positive	20 (13%)	139 (87%)	P <.0001
Negative	63 (34%)	124 (66%)	
**Distance from the Border**			
1 km	39 (98%)	1 (3%)	P <.0001
3 km	3 (9%)	32 (91%)	
12 km	8 (29%)	20 (71%)	
13 km	12 (27%)	33 (73%)	
14 km	21 (11%)	177 (89%)	

**China**			

**Ethnic Minority**			
Yes	28 (13%)	181 (87%)	P = .0053
No	2 (2%)	81 (98%)	
**HIV**			
Positive	8 (17%)	40 (83%)	P = .1242
Negative	22 (9%)	219 (91%)	
**Distance from the Border**			
0 km	20 (51%)	19 (49%)	P <.0001
19 km	3 (17%)	15 (83%)	
57 km	7 (3%)	228 (97%)	

## Pre-publication history

The pre-publication history for this paper can be accessed here:


